# Green Tea Pressurized Hot Water Extract in Atherosclerosis: A Multi-Approach Study on Cellular, Animal, and Molecular Mechanisms

**DOI:** 10.3390/antiox14040404

**Published:** 2025-03-28

**Authors:** Rahni Hossain, Anawat Kongchain, Moragot Chatatikun, Wiyada Kwanhian Klangbud, Chutha Takahashi Yupanqui, Hideyuki J. Majima, Hiroko P. Indo, Pradoldej Sompol, Nazim Sekeroglu, Atthaphong Phongphithakchai, Jitbanjong Tangpong

**Affiliations:** 1 College of Graduate Studies, Walailak University, Nakhon Si Thammarat 80160, Thailand; rahni.ho@mail.wu.ac.th; 2 Department of Medical Technology, School of Allied Health Sciences, Walailak University, Nakhon Si Thammarat 80160, Thailand; anawatbest@gmail.com (A.K.); moragot.ch@wu.ac.th (M.C.); hideyuki.ma@wu.ac.th (H.J.M.); 3 Research Excellence Center for Innovation and Health Products (RECIHP), School of Allied Health Sciences, Walailak University, Nakhon Si Thammarat 80160, Thailand; 4 Medical Technology Department, Faculty of Science, Nakhon Phanom University, Nakhon Phanom 48000, Thailand; wiyadakwanhian@gmail.com; 5 Center of Excellence in Functional Foods and Gastronomy, Faculty of Agro-Industry, Prince of Songkla University, Songkhla 90110, Thailand; chutha.s@psu.ac.th; 6 Department of Oncology, Graduate School of Medical and Dental Sciences, Kagoshima University, Kagoshima City 890-8544, Japan; hindoh@dent.kagoshima-u.ac.jp; 7 Department of Pharmacology & Nutritional Sciences, College of Medicine, University of Kentucky, Lexington, KY 40536, USA; pradoldej.sompol@uky.edu; 8 Department of Biology, Faculty of Arts and Sciences, Gaziantep University, 27310 Gaziantep, Turkey; nsekeroglu@gmail.com; 9 Nephrology Unit, Division of Internal Medicine, Faculty of Medicine, Prince of Songkla University, Songkhla 90110, Thailand; patthaph@medicine.psu.ac.th

**Keywords:** atherosclerosis, oxidized LDL, green tea, *Camellia sinensis*, pressurized hot water extract (GPHWE), apoptosis

## Abstract

Atherosclerosis is a persistent inflammatory disorder influenced by oxidative stress and lipid imbalances, and it continues to be a major contributor to cardiovascular diseases. Rich in catechins and flavonoids, green tea pressurized hot water extract (GPHWE) demonstrated potent antioxidant activity through DPPH, ABTS, hydroxyl, and nitric oxide scavenging assays. In vitro, GPHWE protected RAW264.7 macrophages from oxidized LDL (Ox-LDL)-induced cytotoxicity and apoptosis by mitigating oxidative stress and enhancing cell survival. Animal studies using mice fed a high-fat diet (HFD) revealed notable improvements in lipid profiles, including decreases in total cholesterol, LDL, the atherosclerosis index (AI), the coronary risk index (CRI), and triglycerides, as well as lower levels of malondialdehyde (MDA), an indicator of oxidative stress. These results were comparable to those achieved with Simvastatin. Molecular docking studies indicated strong binding affinities of catechins to essential targets such as LOX-1, HMG-CoA reductase, caspase-3, and Nrf2, implying that the mechanisms of GPHWE involve antioxidant properties, regulation of lipids, and stabilization of plaques. The catechins of GPHWE, including epigallocatechin gallate (EGCG), epicatechin gallate (ECG), and epigallocatechin (EGC), were tentatively identified through qualitative analysis performed by UHPLC-QTOF-MS. This comprehensive approach positions GPHWE as a promising natural remedy for preventing atherosclerosis and reducing cardiovascular risk.

## 1. Introduction

Atherosclerosis, characterized by chronic inflammation and oxidative damage, is the leading cause of cardiovascular diseases (CVDs) such as heart attacks and strokes, which rank among the leading causes of death globally [1]. The progression of atherosclerosis involves a multifaceted process that includes lipid buildup, oxidative stress, and inflammatory reactions [2]. A key event in its advancement is the conversion of low-density lipoprotein (LDL) into oxidized LDL (Ox-LDL), which macrophages absorb to produce foam cells, triggering inflammatory pathways and plaque development [3]. At the same time, an excess of reactive oxygen species (ROS) and reactive nitrogen species (RNS) can overwhelm the antioxidant mechanisms of the body, worsening endothelial dysfunction and cell death [4]. Although traditional treatments like statins successfully reduce cholesterol levels, their drawbacks—such as side effects and insufficient inhibition of atherosclerosis progression—underscore the necessity for alternative treatment options [5].

Green tea (*Camellia sinensis*), abundant in polyphenolic compounds such as catechins, has gained recognition as a potential natural therapeutic agent due to its strong antioxidant, anti-inflammatory, and lipid-lowering effects [6,7]. Among the catechins, epigallocatechin gallate (EGCG), epicatechin gallate (ECG), and epigallocatechin (EGC) have been shown to mitigate oxidative stress, inhibit the uptake of oxidized low-density lipoprotein (Ox-LDL), and diminish plaque formation [8]. However, previous research on green tea catechins has primarily concentrated on in vitro effects or restricted in vivo models, leaving the molecular mechanisms behind their anti-atherosclerotic benefits insufficiently explored. Furthermore, traditional extraction methods frequently compromise bioactive compounds or utilize harmful solvents, which limits their effectiveness [9]. Although the anti-atherosclerotic benefits of green tea are widely recognized, this research notably utilizes pressurized hot water extraction (PHWE) as a sustainable and effective technique, maximizing the retention and bioactivity of catechins without the need for organic solvents. This improves its therapeutic efficacy as a substitute for traditional treatments [10].

This work comprehensively evaluates using a multifaceted strategy to assess the anti-atherogenic potential of green tea pressurized hot water extract (GPHWE). Through antioxidant assays, in vitro cellular experiments, in vivo animal studies, and molecular docking, this research aims to uncover how GPHWE influences oxidative stress, lipid metabolism, and inflammatory pathways. Molecular docking further investigated the interactions between GPHWE polyphenols and specific targets like LOX-1, HMG-CoA reductase, caspase-3, and Nrf2, offering mechanistic insights into its therapeutic effectiveness. Ultra-High-Performance Liquid Chromatography–Quadrupole Time-of-Flight Mass Spectrometry (UHPLC-QTOF-MS) identified polyphenols from GPHWE that interacted strongly with specific target genes. This study presents a novel and sustainable approach to alleviating the burden of atherosclerosis and cardiovascular diseases by combining advanced extraction techniques and individual botanical constituents with molecular and cellular analyses.

## 2. Materials and Methods

### 2.1. Chemicals and Reagents

Folin–Ciocalteu reagent, 2,2-diphenyl-1-picrylhydrazyl (DPPH), 2,2′-azino-bis 3-ethylbenzothi-azoline-6-sulfonic acid (ABTS), gallic acid, quercetin, potassium persulfate, aluminum chloride (AlCl_3_), potassium acetate (CH_3_COOK), sodium carbonate (Na_2_CO_3_), copper sulfate (Cu_2_SO_4_), trichloroacetic acid (TCA), thiobarbituric acid (TBA), phosphate buffer saline (PBS) tablets, dimethyl sulfoxide (DMSO), 3-(4, 5-dimethylthiazol-2-yl)-2, 5-diphenyl-tetrazolium bromide (MTT), RPMI-1640 Medium, L-glutamine, and all chemicals were procured from Sigma-Aldrich (Sigma-Aldrich, St. Louis, MO, USA). Purified water in this study was obtained using a MiliQ system (Millipore, Bedford, MA, USA).

### 2.2. Sample Preparation

Green tea leaves (*Camellia sinensis*) were purchased from Chiang Rai Province in northern Thailand. The leaves were air-dried and ground into small pieces (100 g). In contrast to standard hydrothermal extraction, which involves extended high-temperature treatment, PHWE functions at a refined pressure of 15 psi and a temperature of 100 °C for a brief period of 15 min, maintaining the bioactivity of catechins and reducing oxidative degradation [10]. After cooling to room temperature, the extract was filtered using Whatman No. 1 filter paper and centrifuged at 1500 rpm for 10 min. The resulting supernatant was collected and freeze-dried using a freeze dryer (EYELA Model No: FDU-1200, Tokyo, Japan). GPHWE powder was stored in an airtight container at −20 °C until further analysis.

### 2.3. Determination of Total Phenolic Content

To assess the total phenolic content (TPC), the Folin–Ciocalteu reagent method was utilized, following the procedures described in a prior study [11]. The phenolic content in GPHWE was measured and stated as gallic acid equivalents (GAEs). In summary, 10 μL of GPHWE extracts at different concentrations (ranging from 31.25 to 1000 μg/mL) was placed into a 96-well microplate. Next, 10 μL of Folin–Ciocalteu’s phenol reagent, diluted tenfold, was added to each well and allowed to react for 5 min. Afterward, 100 μL of 7.5% sodium carbonate (Na_2_CO_3_) solution was introduced, and the mixture was incubated in the dark at room temperature for 30 min. The total volume was then adjusted to 200 μL with deionized water, and the absorbance was recorded at 765 nm using a microplate reader (EON, BioTek, VT, USA). Each test was performed in triplicate, with deionized water used as the blank. A calibration curve was created using gallic acid, and the results were reported as mg of gallic acid equivalents per gram of dry weight (mg GAE/g dry weight).

### 2.4. Determination of Total Flavonoid Content

The total flavonoid content (TFC) was analyzed using a modified complex flavonoid-aluminum colorimetric method [12]. In summary, samples of GHPWE at concentrations ranging from 31.25 to 1000 μg/mL, along with standard quercetin solutions created through serial dilutions, were mixed with 100 µL of 2% AlCl_3_ solution and left to incubate for 30 min at room temperature. Afterward, the absorbance was measured at 415 nm using a microplate reader (EON, BioTek, VT, USA) with a blank as the reference. The flavonoid content was calculated and reported in milligrams of quercetin equivalents per gram of dry weight (mg QE/g dry weight). Each measurement was performed in triplicate to ensure accuracy and reliability.

### 2.5. Determination of Free Radical Scavenging Activity on DPPH

The antioxidant properties of the extracts were assessed by their ability to scavenge radicals using the stable radical DPPH [13]. The experimental method, with slight adjustments, was modified from the work of Romanet et al. [14]. In summary, 50 μL of GPHWE at different concentrations was mixed with 200 μL of a 100 μM ethanolic DPPH solution and incubated for 20 min at room temperature away from light. The absorbance of the mixture was then recorded at 517 nm with a microplate reader (EON, BioTek, VT, USA) compared to a blank. The antioxidant capacity of the extract was represented as the inhibitory concentration (IC_50_, μg/mL). All tests were performed in triplicate.

### 2.6. Determination of Free Radical Scavenging Activity on ABTS

The total antioxidant activity of the samples was assessed using the ABTS radical cation decolorization assay [15]. The ABTS radical was generated by mixing a 7 mM ABTS solution with 2.4 mM potassium persulfate and incubating in the dark for 12–18 h. Before the assay, the solution was diluted to an absorbance of 0.700 ± 0.02 at 734 nm. For the evaluation, 20 μL of GPHWE at various concentrations (31.25 to 1000 μg/mL) was mixed with 180 μL of the ABTS•^+^ solution and incubated for 3 min. Absorbance was measured at 734 nm using a microplate reader, and the antioxidant potential was quantified as the inhibitory concentration (IC_50_, μg/mL). All assays were conducted in triplicate.

### 2.7. Hydroxyl Radical Assay

The hydroxyl radical assay followed modified methods based on the Fenton-type reaction [16]. The reaction mixture included 1 mL of 0.1 mM methyl violet, 0.5 mL of 5 mM FeSO_4_, 0.5 mL of 1% H_2_O_2_, and 2 mL Tris buffer (pH 4.0). Next, 20 μL of GPHWE at varying concentrations (31.25 to 1000 μg/mL) or a standard was added to 180 μL of the mixture. Methyl violet served as a trap for the hydroxyl radicals, allowing for decolorization. Absorbance was measured at 565 nm using a microplate reader. The scavenging activity of GPHWE was assessed as the inhibitory concentration (IC_50_, μg/mL), with all experiments performed in triplicate.

### 2.8. Nitric Oxide Scavenging Assay

Nitrite levels were measured using the Griess reagent method, as described in [17]. A mixture of 100 μL sodium nitroprusside (10 mM) and 50 μL GHPWE at concentrations from 31.25 to 1000 μg/mL was incubated for 30 min at room temperature. Afterward, 100 μL of Griess reagent (1% sulfanilamide, 2% O-phosphoric acid, and 0.1% NEDD) was added, and absorbance of the resulting pink chromophore was measured at 546 nm. Nitrite concentration was determined from a standard solution, and percentage inhibition was calculated using the formula [(A_0_ – A_1_)/A_0_] × 100, where A_0_ is the absorbance of the standard, and A_1_ is the absorbance of GHPWE. The scavenging activity on nitrite was expressed as the inhibitory concentration (IC_50_, μg/mL). For accuracy, all experiments were conducted in triplicate.

### 2.9. Cell Culture and Ox-LDL Induced RAW264.7 Macrophage Cell Toxicity

The RAW264.7 macrophage cell line was cultured in RPMI 1640 medium with 4 mM L-glutamine, 1.5 g/L sodium bicarbonate, 4.5 g/L glucose, and 10% fetal bovine serum. Cells were seeded at 1×10^5^ cells per well in 96-well plates and treated with GPHWE, followed by exposure to 100 µg/mL oxidized LDL (Ox-LDL) for 24 h [18]. After replacing the medium, cells were treated with MTT (0.5 mg/mL) for 4 h. Viable cell numbers were determined by formazan production, dissolved in DMSO, and measured at 563 nm, with results compared to untreated controls.

### 2.10. Ox-LDL Induced RAW264.7 Macrophage Cell Apoptosis

Cells were cultured on 6-well plates and treated with Ox-LDL (100 μg/mL) for 24 h, with or without GPHWE (32–256 μg/mL), in a humidified CO_2_ incubator (5% CO_2_). After rinsing twice with PBS, the cells were stained with 0.6 μg/mL Propidium iodide (PI) for 5 min in the dark, followed by three PBS washes. Stained cells were observed under a fluorescence microscope (Zeiss AXIO Vert.) to identify apoptotic cells based on nuclear shrinkage and chromatin condensation [19].

### 2.11. Animals and Study Design

Male IRC mice (*n* = 30), aged 16 weeks, were separated into five groups, each containing six mice. The mice were kept in cages under standard laboratory conditions with a temperature set at 24 °C and a 12 h light/dark cycle, and they had unrestricted access to food and water. The high-fat diet (HFD) was composed of 60% (*w*/*w*) commercial chow, 12% (*w*/*w*) lard oil, 12% (*w*/*w*) sucrose, 8% (*w*/*w*) egg yolk powder, 6% (*w*/*w*) peanut powder, and 1% (*w*/*w*) milk powder [20]. GPHWE was administered orally through gavage at the specified dosage daily at 8:00 am. The experimental protocol received approval from the Animal Ethical Committee of Walailak University (Approval No: WU004/2557).

Group I received a normal chow diet plus distilled water (normal control group).
Group II was given HFD plus distilled water (control group).
Group III was treated with HFD plus GPHWE at 250 mg/kg BW.
Group IV received HFD along with GPHWE at 500 mg/kg BW.
Group V was administered HFD plus Simvastatin at 50 mg/kg BW.

The mice continued on these diets and received GPHWE treatment for eight weeks. Throughout this time, their body weight and food consumption were recorded weekly. After two months on the diets, all mice underwent a 12 h fast before being prepared for blood and tissue sampling. The mice were weighed and then anesthetized using a lethal dose of pentobarbital (65 mg/kg BW), followed by opening their thoracic and abdominal cavities. Blood samples were obtained through cardiac puncture into EDTA tubes, and plasma was separated by centrifugation at 3000 rpm for 10 min. The organs of each mouse were perfused with cold phosphate-buffered saline (PBS, pH 7.4) and were subsequently stored at −80 °C for further analysis.

### 2.12. Blood Analysis for Biochemical Parameters

The plasma samples were utilized for analyzing levels of total cholesterol (TC), triglyceride (TG), high-density lipoprotein cholesterol (HDL-Chol), fasting plasma glucose (FPG), aspartate aminotransferase (AST), alanine aminotransferase (ALT), blood urea nitrogen (BUN), and creatinine. This analysis used an automated chemistry analyzer (Konelab 20i, Thermo Fisher Scientific, MA, USA). Plasma low-density lipoprotein cholesterol (LDL-Chol) was determined using the Friedewald et al. formula [21]. A Risk Marker for Metabolic Syndrome and Cardiovascular Disease Index (MetSCVDI, TG/HDL-Chol), the coronary risk index (CRI, TC/HDL-Chol), and atherogenic index (AI = LDL-Chol/HDL-Chol), robust markers for predicting the risk of atherosclerosis and coronary heart disease, were calculated for all samples [22].

### 2.13. Blood Analysis for Hematological Parameters

Hematological parameters including white blood cell count (WBC), red blood cell count (RBC), hemoglobin concentration (Hb), hematocrit (Hct), mean corpuscular volume (MCV), mean corpuscular hemoglobin (MCH), mean corpuscular hemoglobin concentration (MCHC), and platelet count were determined using a Hematology analyzer (Beckman Coulter, CA, USA).

### 2.14. Determination of Lipid Peroxidation Marker

The levels of MDA in plasma and liver tissue were evaluated as markers of lipid peroxidation using the established protocol by Lovrić et al., with results reported as nM MDA/mL for plasma and nM MDA/g protein for liver tissue, respectively [23]. Lipid peroxidation, indicated by MDA levels, was measured by assessing thiobarbituric acid reactive substances (MDA-TBA). In summary, 150 μL of each sample was mixed with 25 μL of 0.2% BHT and 600 μL of a 15% aqueous solution. The resulting mixture was centrifuged at 4000× *g* for 15 min at 4 °C. Afterward, 300 μL of the deproteinized supernatant was taken and placed into a 2 mL screw cap test tube; then, 600 μL of TBA solution (0.375% in 0.25 M HCl) was added. The samples were then heated at 100 °C for 15 min in boiling water. After cooling, the absorbance of the samples was recorded spectrophotometrically at 535 nm using a BioTeK Synergy H1 Hybrid Reader (BioTek Instruments Inc., Winooski, VT, USA).

### 2.15. Preparation of Sample and LDL Oxidation

LDL was obtained from a hyperlipidemic adult volunteer with a fasting LDL concentration exceeding 200 mg/dL. After an overnight fast, venous blood was collected into clean, sterile glass tubes and allowed to stand for 45 min at room temperature. The serum was separated by centrifuging the tubes at 3000 rpm for 15 min at 4 °C, and the LDL concentration was determined using the Roche COBAS INTEGRA 400 plus analyzer. The concentration of LDL was adjusted to 100 µg/mL and subsequently oxidized in the presence of 40 µM CuSO_4_ at 37 °C for 4 h. As a negative control, LDL at a 100 μg/mL concentration was incubated with PBS. To prevent further oxidation, EDTA was added to achieve a final concentration of 1 mM [24,25]. Approval for the experiment was obtained from the Human Ethical Committee of Walailak University (Approval No: WU067/2559).

### 2.16. Inhibitory Effect of GHPWE on the Cu^2+^-Induced LDL Oxidation

The product of LDL oxidation was assessed by quantifying thiobarbituric acid reactive substances (TBARSs), with minor adjustments to the method outlined by Yagi et al. [26]. LDL samples (500 µg/mL) were pre-treated with various concentrations of GPHWE (32, 64, 128, and 256 μg/mL) for 30 min before being incubated with 200 µM CuSO_4_ for 4 h at 37 °C. For the TBARS assay, protein precipitation was achieved by adding 25% trichloroacetic acid (TCA) to the samples, which were then centrifuged at 10,000 rpm for 10 min at 4 °C. The resulting supernatant was collected and treated with 1% thiobarbituric acid (TBA), followed by heating at 100 °C for 40 min in darkness. Upon completion of the reactions, sample absorbance was measured spectrophotometrically with excitation at 532 nm and emission at 600 nm. The assay was performed in triplicate.

### 2.17. Molecular Docking

The three-dimensional structures of EGCG (CID: 65064), ECG (CID: 107905), and EGC (CID: 72277), which are compounds of GPHWE, were obtained from the PubChem Database [27] (https://pubchem.ncbi.nlm.nih.gov/, accessed on 21 February 2025), accessed on 14 December 2024. The crystal structures of LOX-1 (Lectin-Like Oxidized LDL Receptor 1) (PDB ID: 3R6Y), HMG-CoA reductase (3-Hydroxy-3-Methylglutaryl-Coenzyme A reductase) (PDB ID: 1PKM), Caspase-3 (PDB ID: 3I9I), and Nrf2 (Nuclear Factor Erythroid 2-Related Factor 2) (PDB ID: 2FLU) were downloaded from the Protein Data Bank [28] (http://www.rcsb.org, accessed on 21 February 2025), accessed on 14 December 2024. These target structures underwent optimization and preparation for docking studies using Discovery Studio Visualizer 2020. Molecular docking simulations were performed with AutoDock Vina 1.2.0 [29] (https://vina.scripps.edu/, accessed on 21 February 2025). For each target protein, grid boxes were established to encompass all active binding sites and essential residues. The dimensions of the grid box were set to 126 × 126 × 126 with a grid spacing of 1.000 Å. Ten individual genetic algorithms were executed to generate multiple docking conformations, allowing the selection of the optimal conformation.

### 2.18. Ultra-High-Performance Liquid Chromatography–Quadrupole Time-of-Flight Mass Spectrometry (UHPLC-QTOF-MS) Analysis of GPHWE

The composition of GPHWE was analyzed using UHPLC-QTOF-MS (Agilent TOF/Q-TOF Mass Spectrometer, Agilent Technologies, USA). The instrument was equipped with a Dual AJS electrospray ionization (ESI) source, and data acquisition was performed in the AutoMSMS mode under both positive and negative ionization modes. The separation was conducted on a Zorbax Eclipse Plus C18 Rapid Resolution HD column (150 mm × 2.1 mm, 1.8 µm, Agilent). The mobile phase consisted of 0.1% formic acid in water (A) and acetonitrile with 0.1% formic acid (B), with a gradient elution program, 0 min (100% A), 35 min (10% A, 90% B), and 37 min (100% A), followed by a post-run at 100% A. The flow rate was set at 0.2 mL/min, with an injection volume of 2 µL, and the column temperature was maintained at 25 °C. LC-MS/MS analysis was conducted with an *m*/*z* scanning range of 100 to 1500. The MS parameters included a gas temperature of 350 °C, gas flow of 10 L/min, nebulizer pressure of 60 psig, sheath gas temperature of 275 °C, sheath gas flow of 12 L/min, capillary voltage of 4000 V, nozzle voltage of 2000 V, fragmentor voltage of 175 V, skimmer1 voltage of 65 V, and octopole RF peak of 750 V. Reference masses were set at 121.0509 *m*/*z* and 922.0098 *m*/*z* (positive mode) and 112.9856 *m*/*z* and 1033.9881 *m*/*z* (negative mode). The GPHWE was prepared at 10 mg/mL, filtered through a 0.2 µm nylon membrane, and injected directly into the system. Data processing and compound identification were carried out using MassHunter WorkStation Qualitative Analysis Workflows (V8), METLIN Metabolite Software (V8), and the Personal Compound Database.

### 2.19. Statistical Analysis

The results were expressed as mean ± SEM. All the experiments were performed in triplicates. Statistical data analysis was performed using one-way ANOVA followed by Tukey’s multiple comparison post hoc test (GraphPad Prism 7, San Diego, CA, USA). A *p*-value of less than 0.05 was considered statistically significant.

## 3. Results

### 3.1. Total Phenolic Content, Total Flavonoid Content, and Antioxidant Properties of GPHWE

Table 1 illustrates the evaluation of antioxidants present in GPHWE. It is widely recognized that the total phenolic and flavonoid contents in plant substances function as effective free radical scavengers. GPHWE, extracted at a temperature of 100 °C, under 15 psi, and over a short duration of 15 min, exhibited considerable levels of phenolic and flavonoid content, quantified at 258.61 ± 32.48 mg of gallic acid equivalent per gram of dry weight and 98.91 ± 22.19 mg of quercetin equivalent per gram of dry weight, respectively. The antioxidant capacity of GPHWE was evaluated using ABTS and DPPH scavenging tests, resulting in IC_50_ values of 72.84 ± 4.9 and 66.97 ± 3.73 μg/mL, respectively. Additionally, antioxidant properties of GPHWE involved in vitro scavenging assays targeting OH- and NO radicals provided IC_50_ values of 94.87 ± 8.27 and 77.82 ± 8.04 μg/mL, respectively. The extraction of GPHWE, utilizing highly pressurized hot water for a brief period, displayed impressive total phenolic and flavonoid contents and potent antioxidant and free radical scavenging capabilities.

### 3.2. Ox-LDL Induced RAW264.7 Macrophage Cell Toxicity

**Figure 1:**
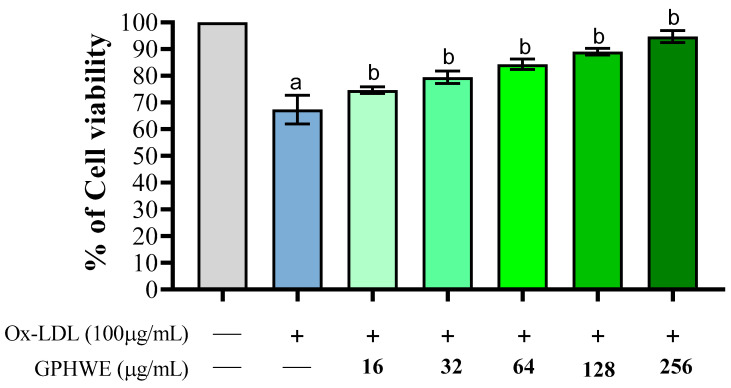
GPHWE inhibited Ox-LDL-induced RAW264.7 macrophage cell toxicity. a: *p* < 0.05 compared between the untreated group and the Ox-LDL group, b: *p* < 0.05 compared between the Ox-LDL group and the Ox-LDL + GHPWE group.

LDL oxidation in the dysfunctional subendothelial space is a key factor in foam cell formation, atheromatous plaque development, and atherosclerotic progression, highlighting the need for effective therapies to prevent its advancement [30]. The impact of Ox-LDL was evaluated regarding its toxicity on RAW264.7 macrophage cells. As shown in Figure 1, Ox-LDL significantly diminished the viability of RAW264.7 macrophage cells (*p* < 0.05). Nevertheless, GPHWE exhibited a protective effect against lipid oxidation in copper-stressed LDL and improved cell viability affected by Ox-LDL. These effects were significantly associated with the concentration of GPHWE in a dose-dependent manner (*p* < 0.05). The results support the potential positive effects of GPHWE in addressing the early stages of atherosclerosis.

### 3.3. Ox-LDL-Induced RAW264.7 Macrophage Cell Apoptosis

**Figure 2:**
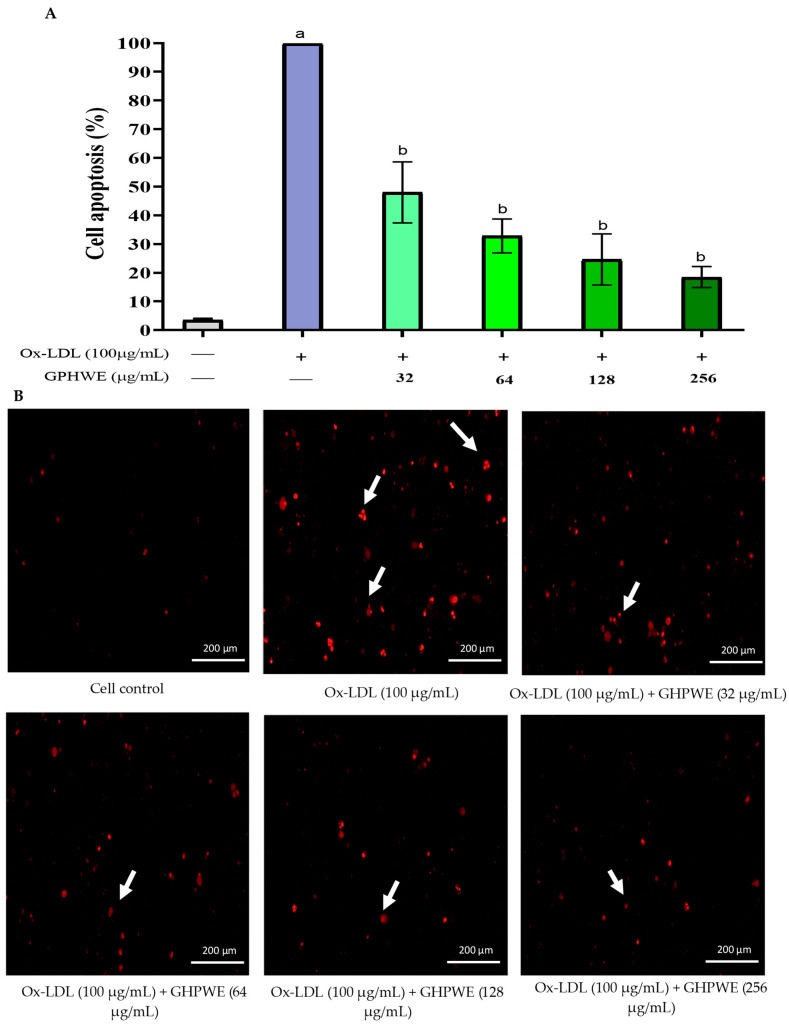
GPHWE inhibited Ox-LDL-induced RAW264.7 macrophage cells. (**A**) Percent of cell apoptosis. (**B**) Nuclear staining of cell apoptosis. a: *p* < 0.05 compared to control, b: *p* < 0.05 compared between Ox-LDL group and GHPWE-co-treated group.

This investigation assessed cell apoptosis by examining nuclear structure changes, specifically nuclear shrinkage and chromatin condensation. RAW264.7 macrophage cells were treated with 100 µg/mL Ox-LDL, with or without varying concentrations of GPHWE for 24 h. Apoptotic cells were identified using PI staining and visualized under a fluorescent microscope. Cells with condensed nuclei appeared bright red, indicating significant apoptosis. A minimum of 200 cells were analyzed [19], revealing increased nuclear condensation in the Ox-LDL group compared to controls (*p* < 0.05). Additionally, GPHWE treatment reduced apoptosis in a dose-dependent manner (*p* < 0.05) (Figure 2A,B).

### 3.4. Animal Characteristics

**Figure 3:**
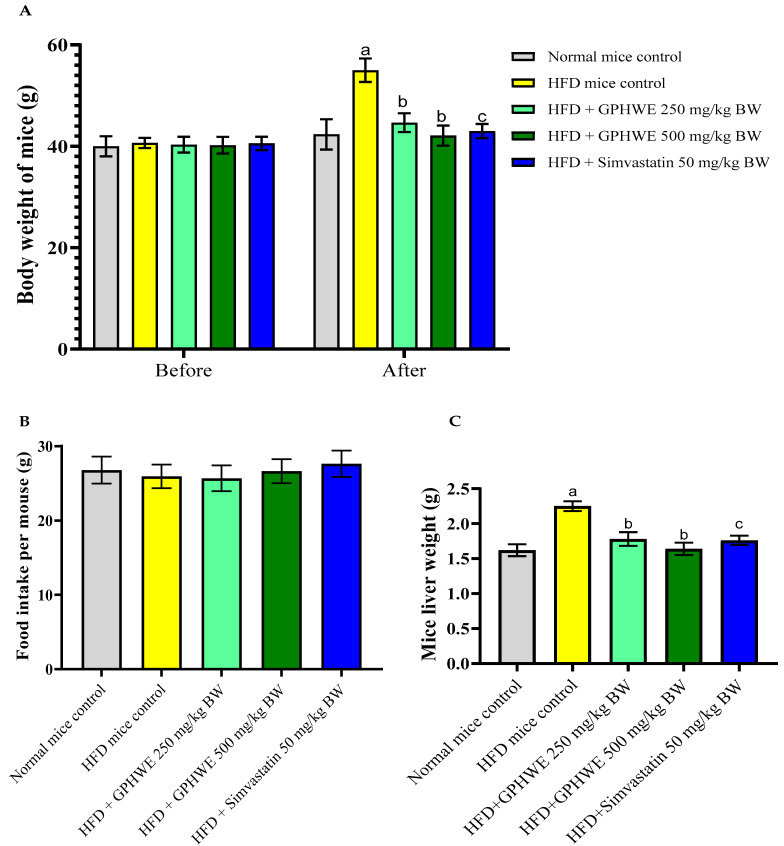
(**A**) Body weight, (**B**) food intake, and (**C**) liver weight of mice fed an HFD and co-treated with GPHWE or Simvastatin for 8 weeks. a: *p* < 0.05 indicates comparison between the normal control group and HFD control group, b: *p* < 0.05 indicates comparison between the HFD control group and the HFD group co-treated with GPHWE, and c: *p* < 0.05 indicates comparison between the HFD control group and the HFD group co-treated with Simvastatin.

Male IRC mice were used to study the effects of an HFD on lipid peroxidation and oxidative damage. Mice were divided into five groups: a normal control fed standard chow, an HFD control group, and two treatment groups (HFD + GPHWE and HFD + Simvastatin) over eight weeks. Results showed that the HFD control group had significant body weight gain compared to the treated groups (*p* < 0.05), as shown in Figure 3A. Food intake data confirmed that the HFD increased weight, but co-treatment reduced it (Figure 3B). The liver tissue weight was significantly higher in the HFD group than in the normal control (*p* < 0.05). At the same time, GPHWE and Simvastatin co-administration reduced liver weight gain significantly (*p* > 0.05), as shown in Figure 3C. The highest liver weight gain was observed in the HFD-only group, with 5% of hepatocytes showing fatty liver features.

**Figure 4:**
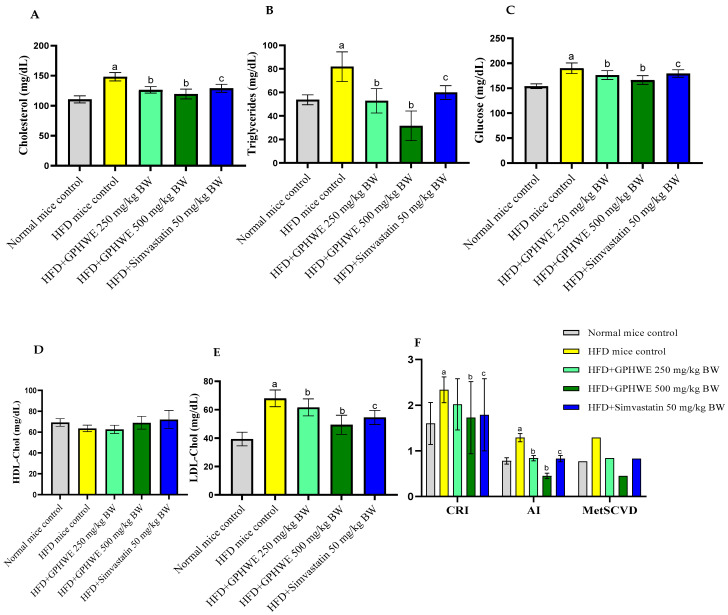
Effect of GPHWE or Simvastatin on lipid and glucose metabolic profiles in HFD-induced mice over 8 weeks. (**A**) Cholesterol, (**B**) triglycerides, (**C**) glucose, (**D**) HDL-cholesterol, (**E**) LDL-cholesterol, and (**F**) CRI = TC/HDL-C), AI = TG/HDL-C, and MetSCVD. a: *p* < 0.05, HFD mice compared to the normal diet control group, b: *p* < 0.05, HFD-treated group compared to HFD + GPHWE, and c: *p* < 0.05, HFD-treated group compared to HFD + Simvastatin. Data are presented as mean ± SEM from three independent experiments. HFD: high-fat diet; GPHWE: green tea pressurized hot water extract; HDL-C: high-density lipoprotein cholesterol; LDL-C: low-density lipoprotein cholesterol; CRI: coronary risk index; AI: atherogenic index.

**Figure 5:**
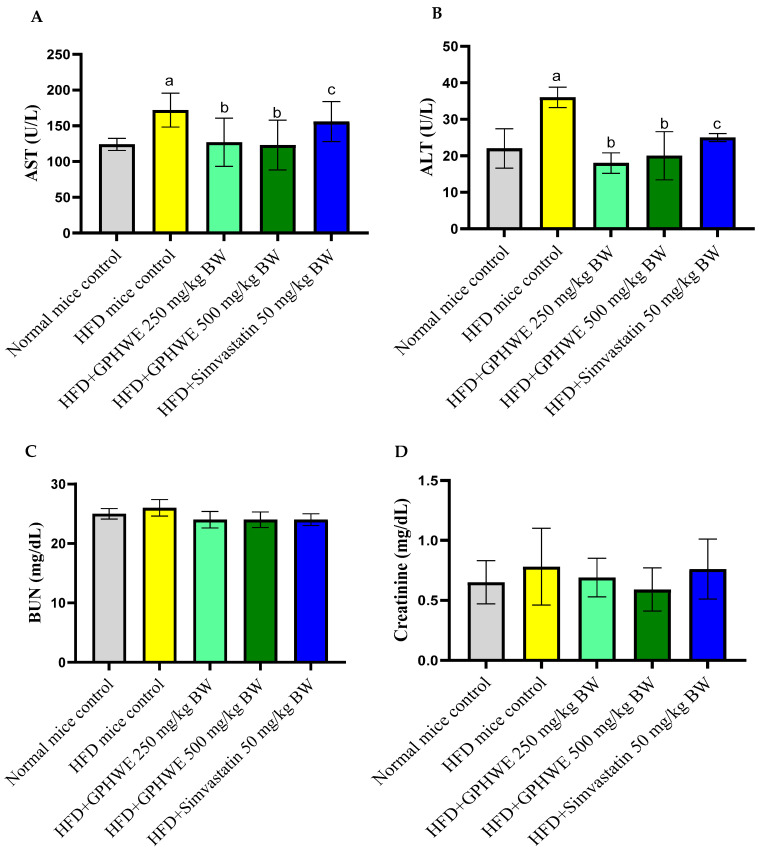
Effect of GPHWE or Simvastatin on liver and kidney function biomarkers in HFD-induced mice over 8 weeks. (**A**) AST, (**B**) ALT, (**C**) BUN, and (**D**) creatinine. a: *p* < 0.05, HFD control group compared to normal diet control group, b: *p* < 0.05, HFD-treated group compared to HFD + GPHWE, and c: *p* < 0.05, HFD-treated group compared to HFD + Simvastatin. Data are presented as mean ± SEM from three independent experiments. HFD: high-fat diet; GPHWE: green tea pressurized hot water extract; AST: aspartate aminotransferase; ALT: alanine aminotransferase; BUN: blood urea nitrogen.

The effects of GPHWE and Simvastatin on biochemical parameters were analyzed in HFD mice over 8 weeks, focusing on their lipid profile characteristics, including total cholesterol, triglycerides, glucose, HDL-Chol, LDL-Chol, AI = TG/HDL-Chol, and CRI = TC/HDL-Chol and MteSCVD (Figure 4A–F). GPHWE significantly improved lipid parameters, reducing total cholesterol, LDL, and triglycerides, while increasing HDL levels, which is comparable to Simvastatin. The reduction in AST and ALT suggests hepatoprotective effects, reinforcing its systemic benefits in lipid metabolism and oxidative stress regulation. Future studies should assess histological changes in arterial plaques to confirm these physiological effects. However, HFD mice treated with GPHWE and Simvastatin showed significantly reduced levels of all parameters (*p* < 0.05). Co-treatment with GPHWE or Simvastatin reduced these enzymes to levels similar to normal chow mice, suggesting that GPHWE may protect the heart and liver without toxicity. BUN and creatinine levels, markers of kidney function, remained consistent across all groups, as shown in Figure 5A–D.

### 3.5. Hematological Parameters

For the hematological assessment of HFD and GPHWE treatment effects, whole blood samples collected with EDTA as an anticoagulant were analyzed. Hematological parameters, including WBCs, RBCs, Hb, Hct, MCV, MCH, MCHC, and platelet counts, showed no statistically significant differences among the groups of HFD mice, various doses of HFD + GPHWE mice, HFD + Simvastatin mice, and those fed standard chow (*p* > 0.05). These findings confirm the safety of GPHWE on hematological parameters, as detailed in Table 2.

### 3.6. Lipid Peroxidation Product in HFD-Treated Mice

**Figure 6:**
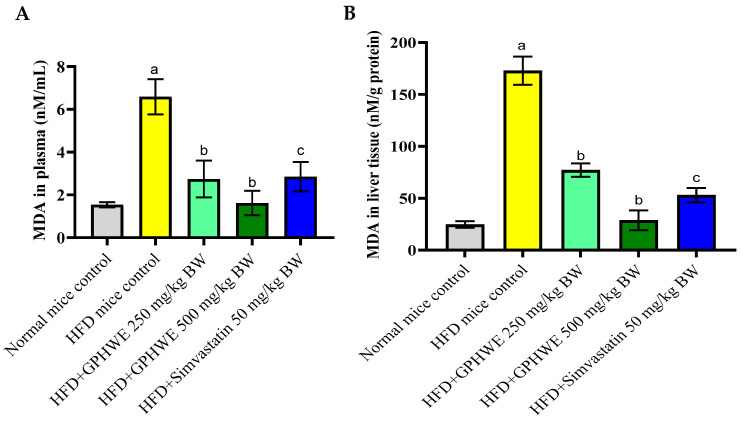
MDA levels in HFD-induced mice and co-treated with GPHWE or Simvastatin for 8 weeks in (**A**) plasma and (**B**) liver tissues. a: *p* < 0.05, HFD control group compared to normal diet control group, b: *p* < 0.05, HFD-treated group compared to HFD + GPHWE, and c: *p* < 0.05, HFD-treated group compared to HFD + Simvastatin.

Figure 6A,B demonstrate a marked elevation in levels of MDA in both plasma and liver tissues of mice that were given an HFD compared to those on a standard chow diet (*p* < 0.05). Nevertheless, the combined treatment with GPHWE or Simvastatin for 8 weeks in mice on the HFD significantly reduced lipid peroxidation in both plasma and liver tissues (*p* < 0.05).

### 3.7. Inhibitory Effect of GPHWE on CuSO_4_-Induced Lipid Peroxidation

**Figure 7:**
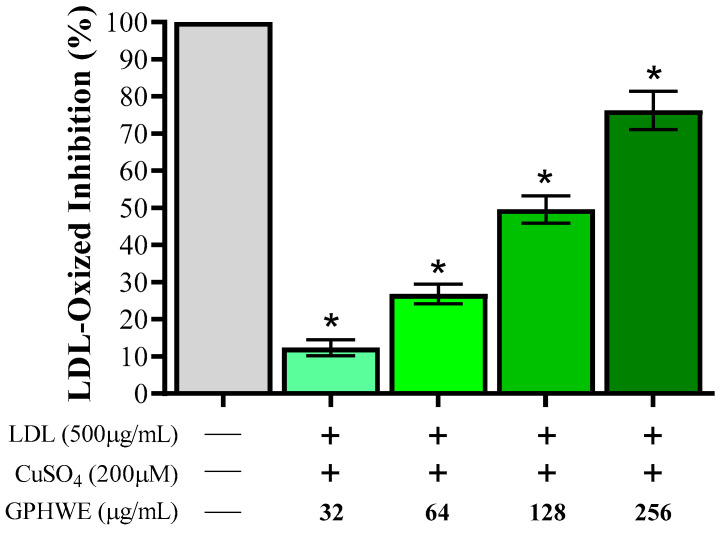
Inhibitory effect of GPHWE on Cu^2+^-induced LDL oxidation. * *p* < 0.05, comparison between the untreated group and the CuSO_4_ + GPHWE group.

The extent of LDL oxidation was evaluated by assessing TBARS levels, with activity quantified using MDA concentration as a marker of lipid peroxidation, compared to a non-oxidized control group. Figure 7 illustrates the inhibitory effect of GPHWE on Cu^2+^-induced LDL lipid peroxidation. These findings demonstrate that GPHWE effectively mitigates lipid peroxidation, with a significant dose-dependent inhibition of Ox-LDL by GPHWE (*p* < 0.05).

### 3.8. Molecular Docking Insights

**Figure 8:**
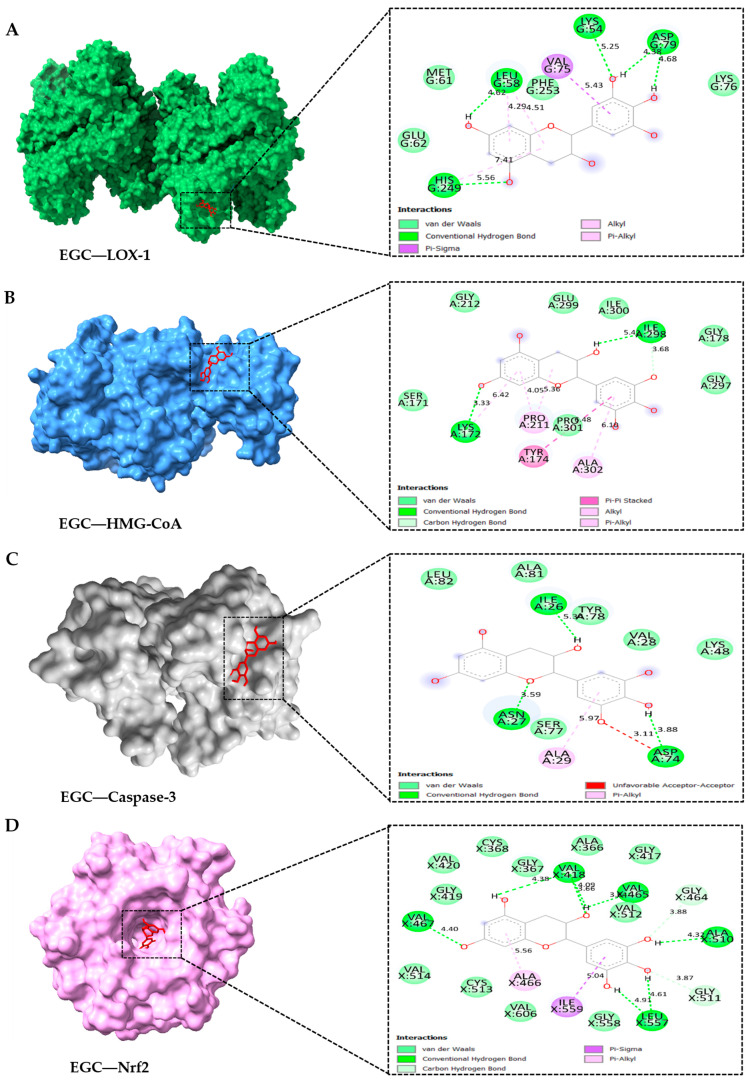
The four best docking results of the GPHWE compound and target enzyme. (**A**) EGC and LOX-1, (**B**) EGC and HMG-CoA reductase, (**C**) EGC and Caspase-3, and (**D**) EGC and Nrf2.

To determine the interaction between anti-atherosclerotic potential agent proteins and green tea catechins, blind docking was completed using AutoDock Vina. Various values shown in Table 3 show how well those proteins and ligands can interact. Binding affinity, hydrogen binding sites, size, and residues were obtained from the results. Affinity value indicates the strength of a ligand binding to the protein and is reported in Kcal/mol. However, the lower the affinity, the stronger the binding interaction between the molecules [31]. The results illustrated in Table 3 show affinity values obtained after binding catechins with the crystal structure. Catechins interacted well with all oncoproteins in the following order: EGC (−3.08 kcal/mol), ECG (−2.60 kcal/mol), and EGCG (−1.69 kcal/mol), for 3R6Y; EGC (−3.30 kcal/mol), ECG (−3.21 kcal/mol), and EGCG (−1.40 kcal/mol), for 1PKM; EGC (−6.95 kcal/mol), ECG (−5.23 kcal/mol), and EGCG (−6.50 kcal/mol), for 3I9I; and EGC (−7.19 kcal/mol), ECG (−6.40 kcal/mol), and EGCG (−5.09 kcal/mol), for 2FLU. Figure 8 presents the best binding energy for the EGC compound of the GPHWE concerning all the target enzymes. Molecular docking revealed strong binding of catechins to key atherosclerosis targets, including LOX-1, HMG-CoA reductase, caspase-3, and Nrf2. Experimental validation through in vitro protein-binding assays and enzyme inhibition studies is necessary to confirm these computational findings. The best-bound positions of catechins with each target are shown, detailing the involved amino acid residues and the types of interactions. Hydrophobic interactions play a key role in these ligand–receptor interactions. [32].

### 3.9. UHPLC-QTOF-MS Analysis of GPHWE

**Figure 9:**
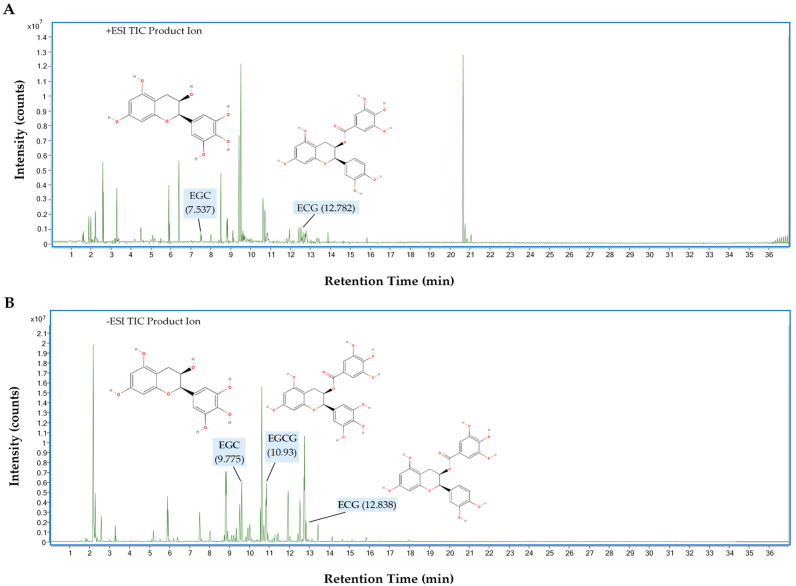
UHPLC-QTOF-MS analysis of GPHWE showing (**A**) positive and (**B**) negative ion modes.

The total ion chromatogram (TIC) from the UHPLC-QTOF-MS analysis of catechins, including EGCG, ECG, and EGC from GPHWE, is shown in Figure 9 for both (A) positive and (B) negative ion modes. Several peaks were observed, with retention times (RTs) ranging from 0.2 to 36 min, molecular mass, and MS/MS fragmentation patterns (Table 4). In the positive ionization mode (+ESI), key peaks for ECG and EGC were observed at *m*/*z* 443.0974 (RT 12.782 min) and 307.0812 (RT 7.537 min). Compounds in the positive ion mode gain protons ([M + H]^+^), forming positively charged species. Some catechins ionize preferentially in this mode, enhancing detection. In the negative ionization mode (−ESI), peaks for EGCG, ECG, and EGC were seen at *m*/*z* 457.0773, 441.0825, and 305.0663, with retention times of 10.93, 12.838, and 9.775 min, respectively. In this mode, compounds ionize by losing protons ([M − H]^−^), resulting in negatively charged species. Certain phenolic compounds, especially catechins, exhibit greater sensitivity in the negative mode, making it ideal for detecting EGCG and ECG. Each chromatogram peak corresponds to a unique compound eluting from the UHPLC system at a specific RT, with values between 7.5 and 12.8 min indicating successful catechin separation in GPHWE. The *m*/*z* differences between +ESI and −ESI modes reveal various ionized forms of the same compounds.

## 4. Discussion

Atherosclerosis results from a complex interaction of oxidative stress, lipid dysregulation, and inflammation. The oxidative modification of Ox-LDL leads to atherogenic particles that are taken up by macrophages, initiating inflammatory responses and contributing to lesions [33]. Elevated cholesterol levels cause accumulation in immune cells, promoting inflammation [34]. Oxidative stress disrupts the balance between antioxidants and free radicals, resulting in cell injury [35]. Macrophages converting Ox-LDL into foam cells produce inflammatory molecules, ultimately leading to apoptosis [36,37]. In this study, GPHWE demonstrated similar effectiveness to Simvastatin in reducing lipid peroxidation and improving lipid profiles, highlighting its potential as a natural supplement for preventing cardiovascular disease compared to conventional green tea extracts and supporting its potential as a novel and sustainable therapeutic agent.

GPHWE showed significant antioxidant activity by scavenging DPPH, ABTS, OH, and NO radicals, which are linked to its high TPC and TFC, especially catechins. It effectively inhibited lipid peroxidation induced by Cu^2+^, thereby preventing the oxidative modification of LDL. Prior studies indicate that catechins can boost total antioxidant capacity and avert LDL oxidation due to their ability to trap radicals and quickly integrate into LDL particles [38,39]. Although this research mainly concentrated on lipid peroxidation and lipid profiles, upcoming studies ought to incorporate biomarkers of inflammation and endothelial dysfunction, like CRP, IL-6, and adhesion molecules, to thoroughly assess the anti-atherosclerotic effects of GPHWE [40,41,42]. Importantly, PHWE technology provides a cost-efficient, quick, and effective method for isolating flavonoid compounds, yielding better recovery rates than techniques like sonication, Soxhlet, and reflux extraction that take 24 h [43]. Additionally, GPHWE can prevent catechin oxidation by polyphenol oxidase enzymes, thereby maintaining the polyphenols in their monomeric forms, similar to the processes seen in tea steaming [44].

Macrophages produce pro-inflammatory molecules like NO, crucial in various inflammatory conditions [45]. Furthermore, phenolic compounds can inhibit NO and peroxynitrite (ONOO−) production [46]. In vitro evaluation using RAW264.7 macrophages demonstrated that GPHWE effectively protected cells from Ox-LDL-induced cytotoxicity in a concentration-dependent manner. Ox-LDL treatment significantly reduced cell viability and increased apoptosis, characterized by nuclear condensation and membrane disruption. GPHWE co-treatment reversed these effects, indicated by fewer apoptotic cells and preserved cell integrity. This protective effect is likely due to the ability of GPHWE to neutralize ROS/RNS and mitigate oxidative damage. The evaluation showed that GPHWE mitigated cell death in RAW264.7 macrophages. Using PI and DNA staining, apoptotic changes were assessed [47]. High ROS/RNS levels from Ox-LDL increased apoptosis [48]. However, GPHWE co-treatment significantly reduced the number of apoptotic cells, suggesting its antioxidant properties may prevent cell apoptosis.

In mice fed an HFD, GPHWE exhibited noteworthy anti-atherogenic effects by enhancing lipid profiles and lowering indicators of oxidative stress. Supplementation with GPHWE reduced total cholesterol, LDL-C, and triglyceride levels while significantly elevating HDL-C levels. This modification in lipid levels resulted in a considerable decrease in the AI and CRI, crucial signs of cardiovascular risk. These results underscore the potential of GPHWE to diminish the likelihood of cardiovascular incidents by addressing lipid imbalance and oxidative stress as significant factors in atherosclerosis [49]. Furthermore, GPHWE lowered MDA levels in plasma and liver tissues, an important marker of lipid peroxidation and oxidative injury [50]. High MDA levels are frequently linked to lipid buildup and the development of atherosclerosis in HFD-induced models [51]. GPHWE showed efficacy by lowering MDA, which helps reduce oxidative stress and lipid peroxidation—both vital for hindering the progression of atherosclerotic lesions [52]. While the TBA spectrophotometric method for MDA determination is commonly used, it has some drawbacks; studies, including Lovrić et al. (2008) [23], indicate it might overestimate MDA levels due to biological interferences. Despite the higher specificity of LC-MS, we opted for the spectrophotometric method to maintain consistency with earlier studies and for its practical accessibility, while recommending LC-MS for future research to improve accuracy. The effectiveness of GPHWE was on par with that of Simvastatin, a commonly used lipid-lowering drug [2]. While Simvastatin mainly inhibits cholesterol synthesis by targeting HMG-CoA reductase, GPHWE seems to produce its effects through a mix of antioxidant functions, prevention of LDL oxidation, and lipid-lowering properties [53]. These combined actions not only enhance lipid metabolism but also safeguard vascular cells from oxidative harm and inflammation, both essential in atherogenesis [54].

The findings from molecular docking offer an additional understanding of the functionality of GPHWE. EGC exhibited the strongest binding affinity toward significant atherosclerosis targets such as LOX-1, HMG-CoA reductase, caspase-3, and Nrf2 [55,56,57,58]. The superior binding of EGC compared to ECG and EGCG is probably attributable to its smaller molecular size and greater flexibility, which facilitate a better fit within the binding sites and enhance hydrogen bonding interactions [59]. By inhibiting LOX-1, GPHWE reduces the uptake of Ox-LDL, while its association with HMG-CoA reductase suggests a potential role in inhibiting cholesterol biosynthesis [60]. Additionally, the interaction with caspase-3 and Nrf2 indicates that GPHWE might aid in preventing macrophage apoptosis and enhancing antioxidant responses, thus stabilizing plaques and decreasing oxidative stress [61].

UHPLC-QTOF-MS analysis provides crucial insights into the catechin composition of GPHWE, confirming the presence of key polyphenols like EGC, ECG, and EGCG, which are known for their potent antioxidant, anti-inflammatory, enzyme inhibition, and neuroprotective effects [62,63]. This qualitative identification strengthens the hypothesis that these compounds contribute to the observed biological effects of GPHWE. Positive and negative ionization modes enhance detection accuracy and ensure comprehensive compound profiling [64]. High-intensity peaks indicate substantial amounts; further quantitative analysis could determine their exact concentrations [65]. Consistent RT values and *m*/*z* ratios demonstrate that the UHPLC-QTOF-MS method is reliable for catechin analysis in GPHWE [66]. EGCG is detected in -ESI but is often absent in the positive ion mode due to ionization efficiency and molecular stability differences [67]. EGCG, with its multiple hydroxyl groups and galloyl moiety, easily loses a proton in -ESI, forming a stable deprotonated ion [63]. In the positive mode, protonation is less favorable, leading to fragmentation and making it harder to detect [66]. This results in a weak or absent EGCG peak in the chromatogram. In contrast, catechins like EGC (without a galloyl group) and ECG (more stable) can ionize more efficiently in both modes [62,66]. Therefore, -ESI is more suitable for detecting EGCG and similar polyphenols, emphasizing the need for both ionization modes in catechin profiling. These findings support the health benefits of GPHWE and inform future pharmacological research. Although we detected catechins in GPHWE using UHPLC-QTOF-MS, their plasma levels were not assessed. Future studies should assess catechin bioavailability to establish a direct connection between their presence and the noted anti-atherosclerotic effects.

This research underscores the advantages of employing PHWE over conventional extraction methods. PHWE is an eco-friendly extraction method that preserves the bioactivity of catechins, avoids toxic solvents, and achieves higher yields more efficiently. Data that compare the efficacy of PHWE in isolating catechins could further bolster its recognition as a sustainable extraction method. Although GPHWE has proven effective in both in vitro and in vivo experiments, further studies, including clinical trials, are necessary to confirm its therapeutic potential in humans. Future research should also explore its long-term safety, bioavailability, and pharmacokinetics to support its transition into clinical application. This study highlights GPHWE as a promising natural remedy for atherosclerosis, exhibiting antioxidant, lipid-lowering, and anti-apoptotic features. Its ability to engage multiple pathways and the environmentally friendly extraction process positions GPHWE as a viable alternative or complementary strategy for preventing cardiovascular diseases.

## 5. Conclusions

This research underscores the potential of GPHWE as a natural supplement for preventing atherosclerosis. It showcases its antioxidant capabilities, lipid-lowering effects, and protective function against oxidative stress-induced cellular damage. The in vivo results indicate that GPHWE is effective at modulating lipid metabolism and reducing the risk of atherosclerosis, with effects similar to Simvastatin. Molecular docking studies support this mechanism by revealing critical interactions between catechins and target proteins related to lipid regulation and oxidative stress. However, some limitations must be recognized. While the study offers mechanistic insights, issues related to catechins’ bioavailability and metabolic stability in physiological conditions remain. Moreover, the findings from animal studies may not directly apply to humans, highlighting the necessity for clinical validation. Future investigations should prioritize long-term safety, pharmacokinetics, and optimal dosing in preclinical and clinical settings and assess potential synergies between GPHWE and current lipid-lowering medications. Our findings position GPHWE as a promising natural adjunct for atherosclerosis prevention, with its antioxidant and lipid-lowering properties likely complementing existing treatments, warranting further clinical exploration. Future studies should aim to optimize extraction methods and validate bioavailability in humans. In summary, this research establishes a solid foundation for advancing GPHWE as a sustainable and effective strategy for cardiovascular disease prevention.

## Figures and Tables

**Table 1:** The antioxidant properties of GPHWE.

Total Phenolic Content (mg GAE/g Dry Weight)	Total Flavonoid Content (mg QE/g Dry Weight)	DPPH Radical Scavenging IC_50_ (μg/mL)	OH- Radical Scavenging IC_50_ (μg/mL)	NO Radical Scavenging IC_50_ (μg/mL)
258.61 ± 32.48	98.91 ± 22.19	72.84 ± 4.90	94.87 ± 8.27	77.82 ± 8.04

Note: Data of mean ± SD of three independent experiments. GAE, gallic acid equivalent; QE, quercetin equivalent; DPPH, 2,2′-diphenyl-1-picrylhydrazyl; OH-, hydroxyl radical; NO, nitric oxide; IC_50_, 50% of inhibitory concentration.

**Table 2:** Effect of HFD-induced mice co-treated with GPHWE or Simvastatin for 8 weeks on hematological parameters.

Group	WBC (cells/µL)	RBC (cells/µL)	Hb (g/dL)	Hct (%)	MCV (fL)	MCH (pg)	MCHC (g/dL)	RDW (%)	Platelet (10^3^/µL)
Normal control	7.42 ± 0.98	8.64 ± 0.67	13.42 ± 0.89	39.68 ± 1.78	54.55 ± 0.98	18.63 ± 0.34	32.08 ± 0.86	15.62 ± 0.47	742.50 ± 167.41
HFD control	8.30 ± 0.59	8.07 ± 0.94	13.08 ± 0.86	39.24 ± 0.85	54.02 ± 0.82	18.23 ± 0.38	31.98 ± 0.98	15.56 ± 0.85	865.41 ± 125.16
HFD + GPHWE250 mg/kg BW	8.02 ± 0.66	8.42 ± 0.74	13.36 ± 0.75	40.06 ± 0.64	54.65 ± 0.58	18.45 ± 0.84	32.06 ± 0.82	15.58 ± 0.67	765.64 ± 132.14
HFD + GPHWE500 mg/kg BW	7.45 ± 0.86	8.74 ± 0.76	13.58 ± 0.59	40.64 ± 0.85	54.83 ± 0.86	18.68 ± 0.96	32.65 ± 0.87	15.82 ± 0.45	763.25 ± 112.35
HFD + Simvastatin50 mg/kg BW	8.12 ± 0.68	8.32 ± 0.65	13.21 ± 0.75	39.62 ± 0.94	54.32 ± 0.87	18.36 ± 0.62	31.88 ± 0.94	15.48 ± 0.84	786.98 ± 154.36

Note: Data are presented as mean ± SEM from three independent experiments. HFD: high-fat diet; GPHWE: green tea pressurized hot water extract; RBC: red blood cell; Hb: hemoglobin; Hct: hematocrit; MCV: mean corpuscular volume; MCH: mean corpuscular hemoglobin; MCHC: mean corpuscular hemoglobin concentration; RDW: red cell distribution width.

**Table 3:** Molecular docking investigations of the phytocompounds of GPHWE with target enzymes of LOX-1, HMG-CoA reductase, Caspase-3, and Nrf2.

Target	Compound	Binding Energy (Kcal/mol)	Hydrogen Bond (Length Å)
LOX-1 (3R6Y)	EGCG	−1.69	ILE-36 (5.67)
	ECG	−2.60	GLY-180 (4.42), ASN-394 (5.78), MET-184 (4.46), LEU-306 (5.13, 6.02)
	EGC	−3.08	LEU-58 (4.62), LYS-54 (5.25), ASP-79 (4.38, 4.68)
HMG-CoA (1PKM)	EGCG	−1.40	LYS-392 (5.82), PHE-25 (5.04), GLU-395 (4.97, 4.25)
	ECG	−3.21	LYS-134 (5.63), THR-138 (3.63), ASN-154 (4.32), GLU-130 (4.18, 4.57)
	EGC	−3.30	LYS-172 (3.33), ILE-298 (5.42)
Caspase-3 (3I9I)	EGCG	−6.50	TRP-41 (5.72, 5.24), LYS-70 (5.85, 6.29), LYS-35 (5.24), GLU-92 (5.27, 6.03), LYS-70 (4.74), GLU-92 (5.59, 5.71, 6.06)
	ECG	−5.23	VAL-122 (2.90), TYR-114 (5.96)
	EGC	−6.95	ASN-27 (3.59), ASP-74 (3.88), ILE-26 (5.34)
Nrf2 (2FLU)	EGCG	−5.09	GLY-81 (4.23), PRO-384 (3.52), SER-383 (4.47, 4.77)
	ECG	−6.40	VAL-561 (3.52), VAL-418 (4.09), VAL-512 (4.79, 4.88), ILE-559 (4.82), VAL-465 (5.12), ARG-326 (6.51)
	EGC	−7.19	VAL-467 (4.40), VAL-418 (3.66, 4.09, 4.38), ALA-510 (4.32), LEU-557 (4.61, 4.91), VAL-465 (3.64)

**Table 4:** UHPLC-QTOF-MS analysis of GPHWE showing the bioactive compounds of catechins.

Retention Time (RT, min)	*M*/*Z*	Observed Mass (g/mol)	Possible Compound	Molecular Formula	Response	Score	Base Peak	Ionization Mode
12.782	443.0974	442.0901	Epicatechin gallate (ECG)	C_22_H_18_O_10_	916,981	49.83	123.0442	Positive (+ESI)
7.537	307.0812	306.0739	Epigallocatechin (EGC)	C_15_H_14_O_7_	536,828	49.48	139.0387	Positive (+ESI)
10.93	457.0773	458.0846	Epigallocatechin Gallate (EGCG)	C_22_H_18_O_11_	4,336,442	98.27	169.0144	Negative (−ESI)
12.838	441.0825	442.0898	Epicatechin gallate (ECG)	C_22_H_18_O_10_	6,574,501	49.19	169.0142	Negative (−ESI)
9.775	305.0663	306.0737	Epigallocatechin (EGC)	C_15_H_14_O_7_	76,723	49.38	196.9156	Negative (−ESI)

## Data Availability

All data analyzed during this study are included in this article.

## Abbreviation

ABTS	2,2′-Azino-bis(3-ethylbenzothiazoline-6-sulfonic acid)
AI	Atherogenic Index
ALT	Alanine Aminotransferase
AST	Aspartate Aminotransferase
BUN	Blood Urea Nitrogen
CRI	Coronary Risk Index
DMSO	Dimethyl Sulfoxide
DPPH	2,2-Diphenyl-1-picrylhydrazyl
ECG	Epicatechin Gallate
EGC	Epigallocatechin
EGCG	Epigallocatechin Gallate
FPG	Fasting Plasma Glucose
GPHWE	Green Tea Pressurized Hot Water Extract
Hb	Hemoglobin
Hct	Hematocrit
HDL-C	High-Density Lipoprotein Cholesterol
HFD	High-Fat Diet
HMG-CoA	3-Hydroxy-3-Methylglutaryl-Coenzyme A
IC50	Inhibitory Concentration (50%)
LDL-C	Low-Density Lipoprotein Cholesterol
LOX-1	Lectin-Like Oxidized LDL Receptor 1
MCH	Mean Corpuscular Hemoglobin
MCHC	Mean Corpuscular Hemoglobin Concentration
MDA	Malondialdehyde
MCV	Mean Corpuscular Volume
MTT	3-(4,5-Dimethylthiazol-2-yl)-2,5-Diphenyltetrazolium Bromide
Nrf2	Nuclear Factor Erythroid 2-Related Factor 2
Ox-LDL	Oxidized Low-Density Lipoprotein
PBS	Phosphate-Buffered Saline
PHWE	Pressurized Hot Water Extraction
QC	Quality Control
QE	Quercetin Equivalent
RDW	Red Cell Distribution Width
SEM	Standard Error of the Mean
TC	Total Cholesterol
TFC	Total Flavonoid Content
TPC	Total Phenolic Content
TG	Triglycerides
UHPLC-QTOF-MS	Ultra-High-Performance Liquid Chromatography–Quadrupole Time-of-Flight Mass Spectrometry
WBC	White Blood Cell
